# Gal-3 is a potential biomarker for spinal cord injury and Gal-3 deficiency attenuates neuroinflammation through ROS/TXNIP/NLRP3 signaling pathway

**DOI:** 10.1042/BSR20192368

**Published:** 2019-12-16

**Authors:** Zhouliang Ren, Weidong Liang, Jun Sheng, Chuanhui Xun, Tao Xu, Rui Cao, Weibin Sheng

**Affiliations:** Department of Spine Surgery, The First Affiliated Hospital of Xinjiang Medical University, Urumqi 830054, Xinjiang Uyghur Autonomous Region, China

**Keywords:** Gal-3, NLRP3, ROS, Spinal cord injury, TXNIP

## Abstract

Spinal cord injury (SCI) often occurs in young and middle-aged population. The present study aimed to clarify the function of Galectin-3 (Gal-3) in neuroinflammation of SCI. Sprague–Dawley (SD) rat models with SCI were established *in vivo*. PC12 cell model *in vitro* was induced by lipopolysaccharide (LPS). Reverse transcription-quantitative polymerase chain reaction (RT-qPCR) and Gene chip were used to analyze the expression levels of genes in the signaling pathway. Histological assessment, ELISA and Western blotting were conducted to evaluate the effects of Gal-3 upon the SCI model. In the *in vivo* SD rat model, Gal-3 expression level was up-regulated. The inhibition of Gal-3 attenuated the neuroinflammation in SCI model. The inhibition of Gal-3 could also mitigate the neuroinflammation and reactive oxygen species (ROS) in *in vitro* model. ROS reduced the effect of Gal-3 on oxidative stress in *in vitro* model. Down-regulating the content of TXNIP decreased the effect of Gal-3 on neuroinflammation in *in vitro* model. Suppressing the level of NLRP3 could weaken the effect of Gal-3 on neuroinflammation in *in vitro* model. Our data highlight that the Gal-3 plays a vital role in regulating the severity of neuroinflammation of SCI by enhancing the activation of ROS/TXNIP/NLRP3 signaling pathway. In addition, inflammasome/IL-1β production probably acts as the therapeutic target in SCI.

## Introduction

Spinal cord injury (SCI) often occurs in young and middle-aged population [1]. Due to the sudden onset of SCI, the mortality and disability rates of SCI are extremely high, causing severe mental stress and heavy economic burden on patients [2]. Studies have shown that although most patients with SCI have an almost-normal life expectancy, they are often burdened with severe motor dysfunction, sensory dysfunction and urinary dysfunction, which greatly decreases their quality of life [1]. Therefore, studies on the pathogenesis and treatment of SCI have attracted accumulative attention.

The pathological process of SCI mainly includes primary and secondary injuries [3]. Because it is difficult to control primary injury, intervention on SCI secondary injury and promotion of nerve function repair have become the study focus [4]. A growing number of studies have shown that innate immune and inflammatory responses are involved in the secondary damage following SCI [3,4]. Massive inflammatory cells mediate inflammatory reactions, leading to functional alterations of various cells and aggravating the death of residual nerve cells following SCI [5].

Inflammasome is a class of macromolecular complexes discovered in recent years, which belongs to the innate immune system [6]. Activation of inflammasomes can cleave pro-IL-1β and pro-IL-18 to produce active forms of IL-1β and IL-18, causing a series of downstream inflammatory reactions. In addition, inflammasome can also regulate caspase-1 and induce programmed cell death under pathological conditions [6,7].

TXNIP is an important oxidative stress factor in the body, which has been mostly studied in myocardial ischemia and cerebral ischemia. It has been reported that TXNIP can bind to NLRP3 inflammasome in a diseased state to activate NLRP signaling pathway, thereby causing brain infarction by activating inflammatory response [8,9]. At present, the TXNIP/NLRP3 signaling pathway has been studied in SCI, revealing that inhibition of the above pathway could protect mice from SCI [9,10].

Galectin-3 (Gal-3), a member of the galectin family, contains a sugar recognition domain with a specific affinity for β-galactoside, which is widely distributed in the nucleus, cytoplasm, cell surface and cell matrix [11]. Gal-3, a potent inflammatory signaling molecule, promotes coronary inflammatory response, promotes the phagocytosis of low-density lipoproteins by monocytes, macrophages and vascular smooth muscle cells, promotes the proliferation of foam cells, binds with terminal glycosylation product-modified lipids, promotes the deposition of lipids on the vascular wall to form atheromatous plaques, aggravates local proteolysis, plaque rupture and thrombosis, thereby causing myocardial ischemia [12,13]. The present study was designed to clarify the function of Gal-3 in neuroinflammation of SCI.

## Materials and methods

### Animal model establishment

The present study was approved by the Scientific Review Committee and the Institutional Review Board of the First Affiliated Hospital of Xinjiang Medical University. The animal experiment was conducted in the Animal Laboratory of the First Affiliated Hospital of Xinjiang Medical University.

Sprague–Dawley (SD) rats (170–190 g) were fed with standard animal feed and then anesthetized with 35 mg/kg pentobarbital sodium. Rats were fixed on the operating table and T9 spinous processes were identified. Subcutaneous tissues in this area were incised along the posterior median line. T8–T9 spinous processes and lamina were exposed. T8 and T9 spinous processes and lamina were removed, right side of the spinal cord was cut and right hindlimb was considered to indicate a successful model of SCI. All rats were randomly distributed into control and SCI model groups (*n*=6/every group). Next, SCI rats (*n*=6) were treated with 10 mg/kg of GB1107 (p.o) for 24 h.

### Histological assessment

The spinal cord tissues were fixed in 4% formalin for a minimum of 24 h and tissues were embedded in paraffin and sectioned (5 µm) for staining. Spinal cord tissue was stained with H.E staining and was observed via an optical microscope (BX-42, Olympus Corporation, Tokyo, Japan).

### Reverse transcription-quantitative polymerase chain reaction

Total RNA was isolated from the spinal cord tissue and cells using TRIzol reagent (Invitrogen, CA, U.S.A.) according to manufacturer’s protocol. TaqMan MicroRNA Reverse Transcription kit (Life Technologies) was then used to synthesize cDNA. Reverse transcription-quantitative polymerase chain reaction (*RT-qPCR*) was performed using SYBR-Green Universal qPCR Master Mix (Bio-Rad, Hercules, CA, U.S.A.) by an ABI 7500 thermocycler (Life Technologies). The thermal cycling conditions were set as 95°C for 10 min, followed by 40 cycles at 95°C for 30 s and 60°C for 30 s. Gene expression was determined by 2^−ΔΔ*C*_t_^.

### Gene chip assay

The RNA quality and quantity were measured by Agilent Bioanalyzer 2100 (Agilent Technologies, Santa Clara, CA, U.S.A.). The sign analysis were performed by Agilent Microarray Scanner (Cat # G2565CA, Agilent Technologies, Santa Clara, CA, U.S.A.).

### Cell culture and transfection

PC12 cells were purchased from Shanghai Cell Bank of Chinese Academy of Sciences (Shanghai, China) and cultured in Dulbecco’s modifed Eagle’s medium (DMEM, Life Technologies, Carlsbad, CA, U.S.A.) with 10% fetal bovine serum (FBS; Life Technologies, Carlsbad, CA, U.S.A.) at 37°C in 5% CO_2_. PC12 cells were transfected with si-Gal-3 or negative mimics using Lipofectamine 2000. After 48-h transfection, PC12 cells were cultured with 100 ng/ml lipopolysaccharide (LPS) for 4 h. PC12 cells were cultured with 100 ng/ml LPS and NAC (2 mM) for 4 h. Next, PC12 cells were transfected with si-Gal-3 or negative mimics using Lipofectamine 2000. After 48-h transfection, PC12 cells were cultured with 100 ng/ml LPS and H_2_O_2_ (1 mM) for 4 h. PC12 cells were transfected with Gal-3 plasmid or negative mimics using Lipofectamine 2000. After transfection for 48 h, PC12 cells were cultured with 100 ng/ml LPS and NAC (2 mM) for 4 h. PC12 cells were transfected with si-TXNIP+Gal-3 plasmid or negative mimics using Lipofectamine 2000. After transfection for 48 h, PC12 cells were cultured with 100 ng/ml LPS for 4 h. Subsequently, PC12 cells were transfected with TXNIP+si-Gal-3 plasmid or negative mimics using Lipofectamine 2000. After transfection for 48 h, PC12 cells were cultured with 100 ng/ml LPS for 4 h.

### ELISA

NF-KB, p65, TNF-α, IL-1β, IL-6, MDA, SOD, CAT and GSH-PX levels were measured using ELISA kits (Beyotime Institute of Biotechnology). The optical density (OD) was measured using a Multiskan FC enzyme immunoassay analyzer (Thermo Fisher Scientifc, Waltham, MA, U.S.A.) at a wavelength of 450 nm.

### Western blotting

The tissues and cells’ samples were solubilized in RIPA lysis at 4°C and the protein was quantified using BCA assay; 50 µg protein was separated with 10% sodium dodecyl sulfate/polyacrylamide gel electrophoresis (SDS/PAGE), and transferred on to a polyvinylidene difluoride membrane. Membrane was blocked with 5% non-fat in TBST for 1 h at 37°C and incubated with Gal-3 (1:500, Cell Signaling Technology), TXNIP (1:500, Cell Signaling Technology), NLRP3 (1:500, Cell Signaling Technology) and GAPDH (1:2000, Cell Signaling Technology) at 4°C overnight. Membrane was washed with TBST and incubated with goat anti-rabbit monoclonal IgG (1:10000; Cell Signaling Technology) secondary antibodies at room temperature for 1 h. Membrane was visualized with an ECL kit (Pierce Chemical Co.) and analyzed by Image-Pro Plus 6.0 software.

### Immunofluorescent staining

Cells were fixed with 4% polyformaldehyde for 20 min, blocked with 5% BSA in PBS and 0.2% Triton X-100 for 30 min. Cells were incubated with Gal-3 (1:100, Cell Signaling Technology) at 4°C overnight. Cells were incubated with goat anti-rabbit IgG-CFL 555 (1:100, Santa Cruz Biotechnology) at 37°C for 2 h after washing with PBST for 15 min and subsequently stained with DAPI in dark room for 15 min and washed with PBST for 15 min. Cells were analyzed using the Olympus BX51 fluorescence microscope.

### Statistical analysis

All data were normally distributed and were expressed as mean ± SD. Statistical significance was assumed at *P*<0.05. Statistical significance was determined by independent-sample Student’s *t* test or ANOVA.

## Results

### Gal-3 expression in SCI model

**Figure 1:**
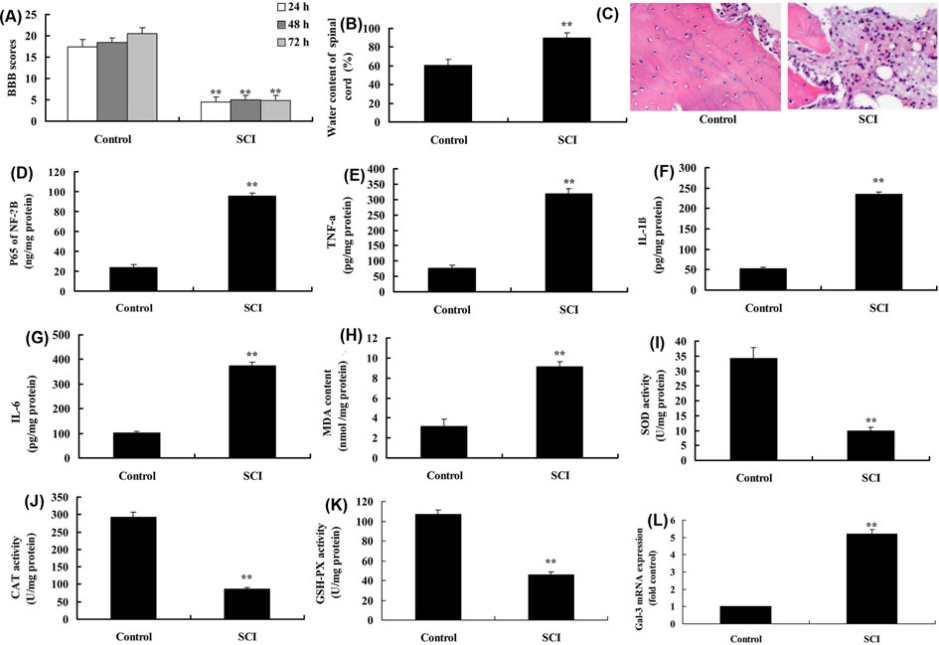
Gal-3 expression in SCI model BBB score (**A**), water content of spinal cord (**B**), HE staining (**C**), p65 (**D**), TNF-α (**E**), IL-1β (**F**), IL-6 (**G**), MDA (**H**), SOD (**I**), CAT (**J**) and GSH-PX levels (**K**), Gal-3 mRNA expression (**L**) in SCI model. Control, control sham group; SCI, SCI model group. **P<0.01 compared with control group.

BBB score was reduced, whereas the water content of spinal cord was increased in SCI model, compared with sham group (Figure 1A,B). Spinal cord cells were damaged and the number of spinal cord cells was reduced in SCI model, compared with sham group (Figure 1C). In addition, p65, TNF-α, IL-1β, IL-6 and MDA levels were increased, whereas SOD, CAT and GSH-PX levels were reduced in SCI model, compared with sham group (Figure 1D–K). At last, the expression of Gal-3 mRNA was up-regulated in SCI model compared with that in sham group (Figure 1L).

### Inhibition of Gal-3 attenuates neuroinflammation in SCI model

**Figure 2:**
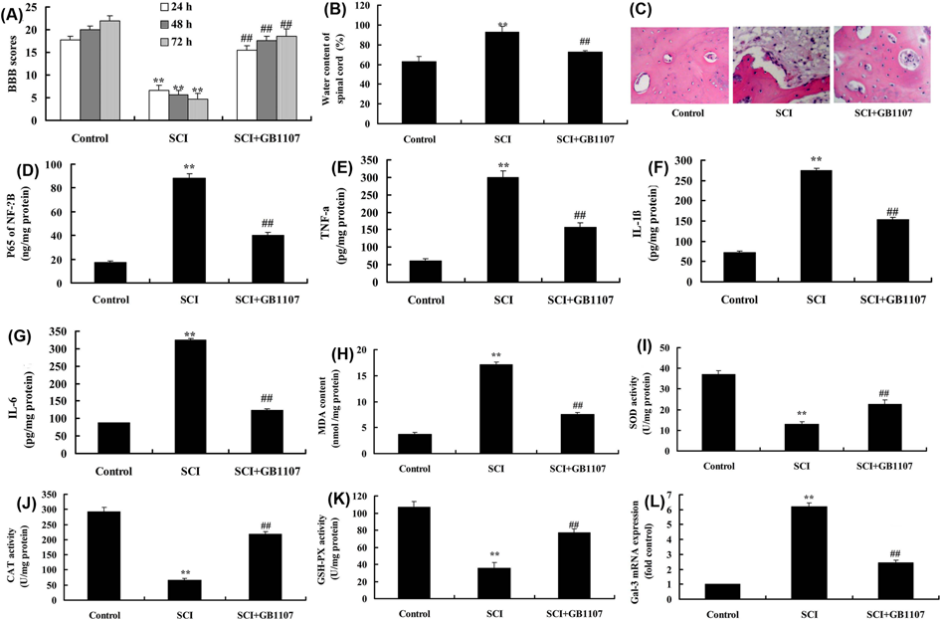
The inhibition of Gal-3 attenuates neuroinflammation in SCI model BBB score (**A**), water content of spinal cord (**B**), HE staining (**C**), p65 (**D**), TNF-α (**E**), IL-1β (**F**), IL-6 (**G**), MDA (**H**), SOD (**I**), CAT (**J**) and GSH-PX levels (**K**), Gal-3 mRNA expression (**L**) in SCI model. Control, control sham group; SCI, SCI model group; SCI+GB1107, SCI model by GB1107 group. **P<0.01 compared with control group, ^##^*P*<0.01 compared with SCI model group.

Compared with SCI model, Gal-3 inhibitor (10 mg/kg, p.o), GB1107 increased BBB score, reduced water content of spinal cord, restored the number of spinal cord cells in SCI model (Figure 2A–C). Besides, GB1107 suppressed the expression level of Gal-3 mRNA, p65, TNF-α, IL-1β, IL-6 and MDA, whereas it up-regulated the SOD, CAT and GSH-PX levels in SCI model, compared with those in SCI model (Figure 2D–L).

### Gal-3 targets TXNIP/NLRP3

**Figure 3:**
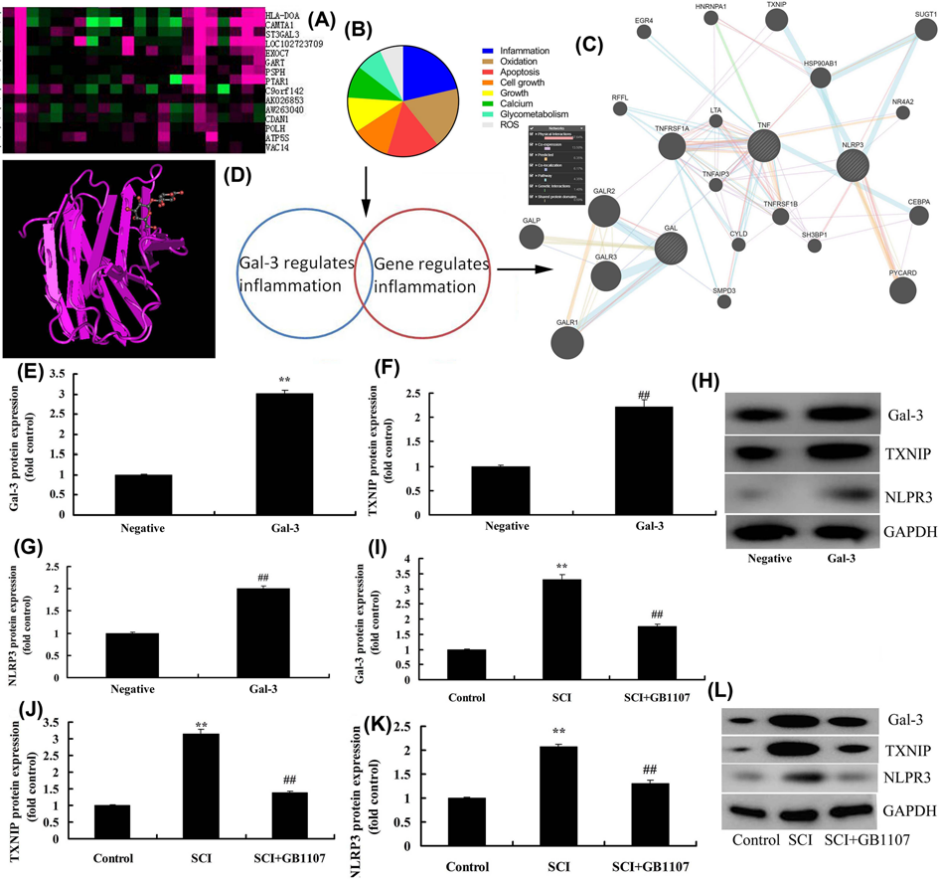
Gal-3 target spot TXNIP/NLRP3 Gene chip (**A**), analysis chart (**B**), network signal diagram (**C**), structural formula of Gal-3 (**D**), Gal-3 induced Gal-3, TXNIP and NLRP3 protein expressions in *in vitro* model of SCI (**E**–**H**), GB1107 suppressed Gal-3, TXNIP and NLRP3 protein expressions in rat model of SCI (**I**–**L**). Negative, negative mimic group; Gal-3, overexpression of Gal-3 group; Control, control sham group; SCI, SCI model group; SCI+GB1107, SCI model by GB1107 group. **P<0.01 compared with negative or control group, ^##^*P*<0.01 compared with SCI model group.

To further investigate the mechanism of Gal-3 in SCI model, Gene chip was performed to analyze the target of Gal-3. Gal-3 could affect the inflammation and oxidation apoptosis etc. Target point and inflammation gene was used to analyze the underlying mechanism. These results demonstrated that TXNIP/NLRP3 may be the signaling pathway for Gal-3 in SCI (Figure 3A–C). Structural formula of Gal-3 was illustrated in Figure 3D. Overexpression of Gal-3 could up-regulate the expression of Gal-3, TXNIP and NLRP3 proteins in *in vitro* model of SCI, compared with negative group (Figure 3E–H). GB1107 down-regulated the expression levels of Gal-3, TXNIP and NLRP3 proteins in SCI rat model (Figure 3I–L).

### Inhibition of Gal-3 attenuates neuroinflammation and reactive oxygen species in *in vitro* model

**Figure 4:**
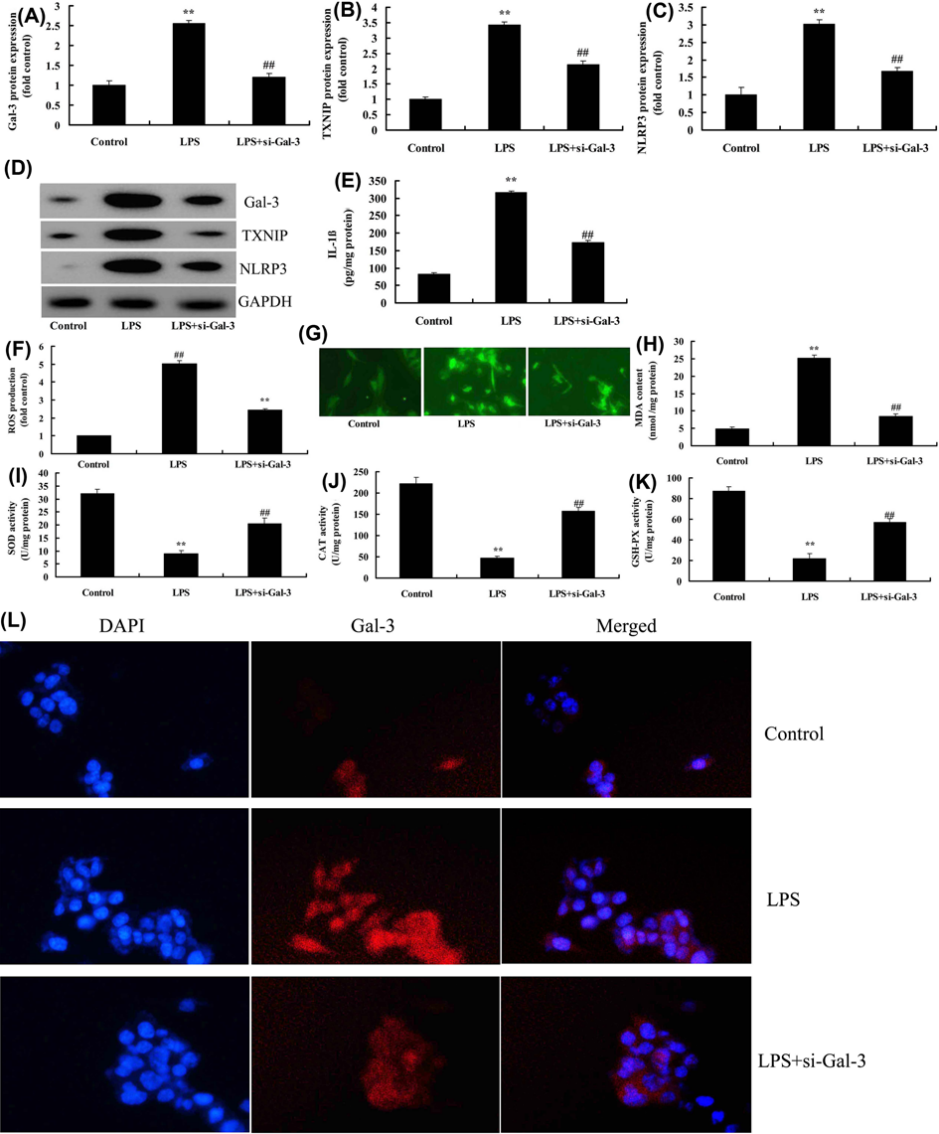
The inhibition of Gal-3 attenuates neuroinflammation and ROS in *in vitro* model Gal-3, TXNIP and NLRP3 protein expressions (**A**–**D**), IL-1β levels (**E**), ROS levels (**F,G**), MDA (**H**), SOD (**I**), CAT (**J**) and GSH-PX levels (**K**), Gal-3 protein expression (immunofluorescent staining, (**L**). Control, control group; LPS, SCI model group; LPS+si-Gal-3, SCI model by si-Gal-3 group. **P<0.01 compared with control group, ^##^*P*<0.01 compared with SCI model group.

To evaluate the effects of Gal-3 upon the *in vitro* SCI model, PC12 induced by LPS was treated with si-Gal-3. Si-Gal-3 suppressed the expression levels of Gal-3, TXNIP and NLRP3 proteins and down-regulated IL-1β levels in *in vitro* model, compared with LPS model group (Figure 4A–E). The si-Gal-3 reduced the reactive oxygen species (ROS) and MDA levels, whereas increased SOD, CAT and GSH-PX levels in *in vitro* model, compared with LPS model group (Figure 4F–K). Immunofluorescent staining showed that si-Gal-3 down-regulated the Gal-3 expression in *in vitro* model, compared with LPS model group (Figure 4L).

### ROS regulates the effect of Gal-3 on oxidative stress in *in vitro* model

**Figure 5:**
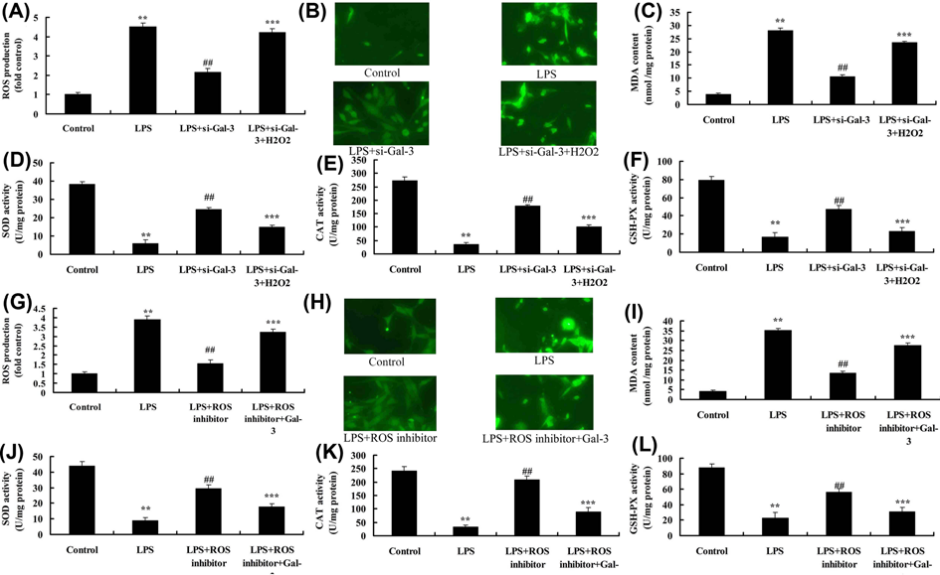
ROS regulates the effects of Gal-3 on oxidative stress *in vitro* model ROS levels (**A,B**), MDA (**C**), SOD (**D**), CAT (**E**) and GSH-PX levels (**F**) *in vitro* model by overexpression of ROS; ROS levels (**G,H**), MDA (**I**), SOD (**J**), CAT (**K**) and GSH-PX levels (**L**) *in vitro* model by ROS inhibitor. Control, control group; LPS, SCI model group; LPS+si-Gal-3, SCI model by si-Gal-3 group; LPS+si-Gal-3+H_2_O_2_, SCI model by si-Gal-3 and H_2_O_2_ group; LPS+ROS inhibitor, SCI model by ROS inhibitor group; LPS+ROS inhibitor+Gal-3, SCI model by ROS inhibitor and Gal-3 group. **P<0.01 compared with control group, ^##^*P*<0.01 compared with SCI model group; ***P<0.01 compared with LPS+ROS inhibitor or LPS+si-Gal-3 group.

To determine the role of ROS in the effects of Gal-3 on oxidative stress in *in vitro* model, H_2_O_2_ was supplemented into cells by LPS and si-Gal-3. H_2_O_2_ increased ROS and MDA levels, and reduced SOD, CAT and GSH-PX levels in LPS and si-Gal-3 group, compared with LPS and si-Gal-3 group (Figure 5A–F). ROS inhibitor reduced ROS and MDA levels, and promoted SOD, CAT and GSH-PX levels in LPS group, compared with LPS group (Figure 5G–L). Conversely, overexpression of Gal-3 increased ROS and MDA levels, whereas reduced SOD, CAT and GSH-PX levels in LPS+ROS inhibitor group, compared with LPS+ROS inhibitor group (Figure 5G–L).

**Figure 6:**
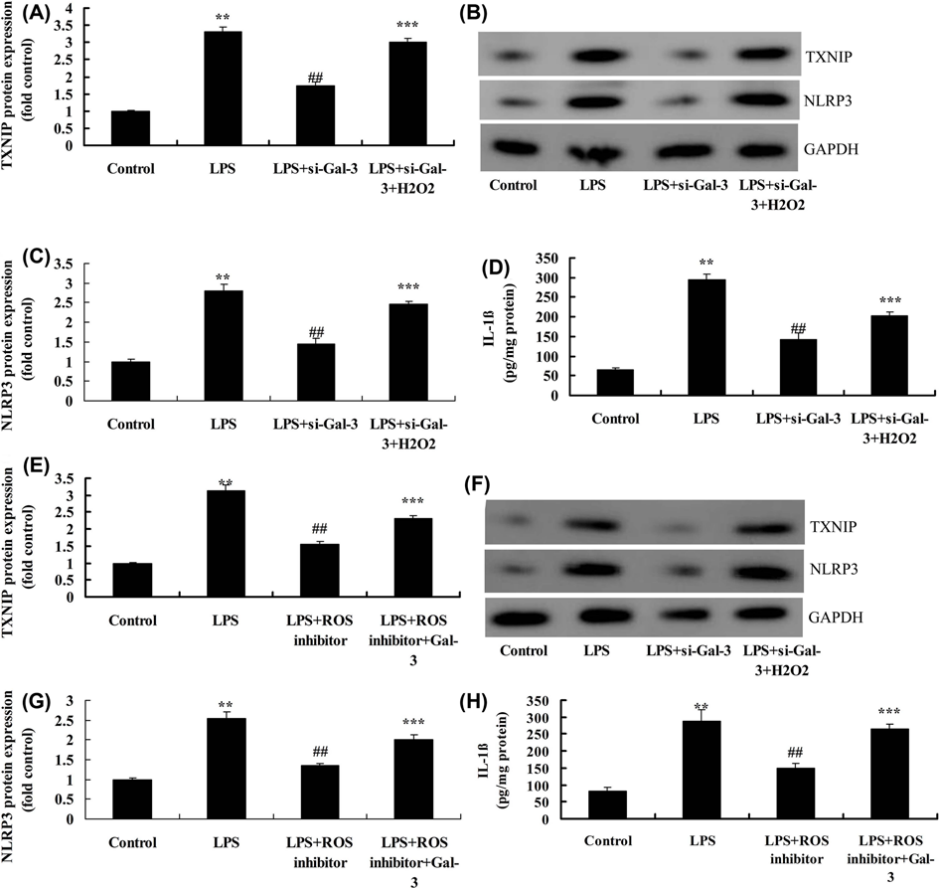
ROS regulates the effects of Gal-3 on neuroinflammation *in vitro* model Expression of TXNIP and NLRP3 proteins (**A**–**C**), IL-1β levels (**D**) by overexpression of ROS; TXNIP and NLRP3 protein expressions (**E**–**G**), IL-1β levels (**H**) by ROS inhibitor. Control, control group; LPS, SCI model group; LPS+si-Gal-3, SCI model by si-Gal-3 group; LPS+si-Gal-3+H_2_O_2_, SCI model by si-Gal-3 and H_2_O_2_ group; LPS+ROS inhibitor, SCI model by ROS inhibitor group; LPS+ROS inhibitor+Gal-3, SCI model by ROS inhibitor and Gal-3 group. **P<0.01 compared with control group, ^##^*P*<0.01 compared with SCI model group; ***P<0.01 compared with LPS+ROS inhibitor or LPS+si-Gal-3 group.

H_2_O_2_ also up-regulated the expression of TXNIP and NLRP3 proteins and IL-1β in LPS and si-Gal-3 group, compared with LPS and si-Gal-3 group (Figure 6A–D). Next, ROS inhibitor suppressed the expression of TXNIP and NLRP3 proteins, and reduced IL-1β levels in LPS group, compared with LPS group (Figure 6E–H). Overexpression of Gal-3 induced the expression levels of TXNIP and NLRP3 proteins, and increased IL-1β levels in LPS+ROS inhibitor group, compared with LPS+ROS inhibitor group (Figure 6E–H).

### Inhibition of TXNIP reduced the effect of Gal-3 on neuroinflammation in *in vitro* model

**Figure 7:**
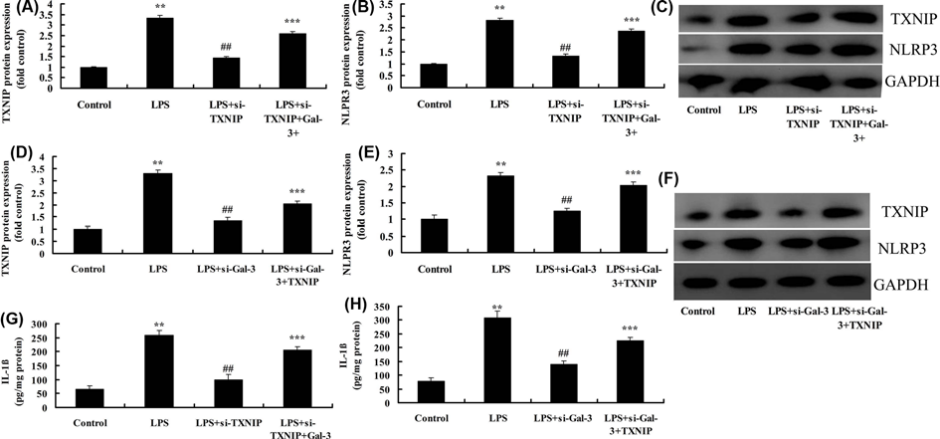
The inhibition of TXNIP reduced the effect of Gal-3 on neuroinflammation *in vitro* model TXNIP and NLRP3 protein expressions (**A**–**C**) by overexpression of TXNIP; TXNIP and NLRP3 protein expressions (**D**–**F**) by down-regulation of TXNIP; IL-1β levels (**G**) by overexpression of TXNIP; IL-1β levels (**H**) by down-regulation of TXNIP. Control, control group; LPS, SCI model group; LPS+si-TXNIP, SCI model by si-TXNIP group; LPS+si-TXNIP+Gal-3, SCI model by si-TXNIP+Gal-3 group; LPS+si-Gal-3, SCI model by i-Gal-3 group; LPS+si-Gal-3+TXNIP, SCI model by si-Gal-3+TXNIP group. **P<0.01 compared with control group, ^##^*P*<0.01 compared with SCI model group; ***P<0.01 compared with LPS+si-TXNIP and LPS+si-Gal-3 group.

Si-TXNIP suppressed the expression of TXNIP and NLRP3 proteins, and reduced IL-1β levels in LPS group, compared with LPS group (Figure 7A–C,G). Overexpression of Gal-3 induced the expression of TXNIP and NLRP3 proteins, and increased IL-1β levels in LPS+si-TXNIP group, compared with LPS+si-TXNIP group (Figure 7A–C,G). Next, overexpression of TXNIP induced the expression of TXNIP and NLRP3 proteins, and increased IL-1β levels in LPS+si-Gal-3 group, compared with LPS+si-Gal-3 group (Figure 7D–F,H). These results showed that TXNIP participated in the effect of Gal-3 on neuroinflammation in *in vitro* model of SCI.

### Inhibition of NLRP3 reduced the effect of Gal-3 on neuroinflammation in *in vitro* model

**Figure 8:**
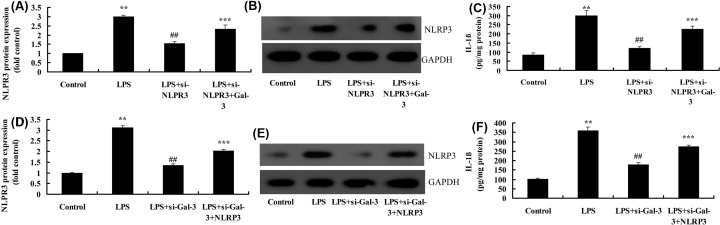
The inhibition of NLRP3 reduced the effect of Gal-3 on neuroinflammation *in vitro* model NLRP3 protein expressions (**A,B**), IL-1β levels (**C**) by overexpression of NLRP3; NLRP3 protein expressions (**D,E**), IL-1β levels (**F**) by down-regulation of NLRP3; Control, control group; LPS, SCI model group; LPS+si-NLRP3, SCI model by si-NLRP3 group; LPS+si-NLRP3+Gal-3, SCI model by si-NLRP3+Gal-3 group; LPS+si-Gal-3, SCI model by i-Gal-3 group; LPS+si-Gal-3+ NLRP3, SCI model by si-Gal-3+ NLRP3 group. **P<0.01 compared with control group, ^##^*P*<0.01 compared with SCI model group; ***P<0.01 compared with LPS+si-NLRP3 and LPS+si-Gal-3 groups.

Si-NLRP3 down-regulated the expression of NLRP3 protein and reduced IL-1β levels in *in vitro* model, compared with *in vitro* model group (Figure 8A–C). However, overexpression of Gal-3 up-regulated the expression of NLRP3 protein and increased IL-1β levels in *in vitro* model by si-NLRP3, compared with *in vitro* model by si-NLRP3 group (Figure 8A–C). Overexpression of NLRP3 up-regulated the expression of NLRP3 protein and IL-1β levels in *in vitro* model by si-Gal-3, compared with *in vitro* model si-Gal-3 group (Figure 8D–F). These experimental data indicated that Gal-3 aggravated the neuroinflammation of SCI via NLRP3.

## Discussion

SCI is a common clinical disease, which occurs mostly in young adults. SCI is clinically characterized by high morbidity, high disability and high mortality rate, which would not only cause serious damage to family and the body, but also brings heavy economic burden to the whole society [14,15]. Therefore, in-depth elucidation of the pathogenesis of SCI has always been a hot and difficult point in the global medical research [14]. In the present study, we addressed that Gal-3 mRNA expression in SCI model was up-regulated, compared with sham group. Mendonça et al. [16] provided evidence that lack of Gal-3 reduced neuroinflammation and protected the retina and optic nerve in diabetic mice.

Gal-3, an important member of the lectin family, is mainly distributed in tumor cells, epithelial cells, macrophages and inflammatory cells, which is involved in multiple types of biological functions, including regulating inflammation, regulating cell growth, anti-apoptosis and mediating cell adhesion [17]. Under different conditions, the biological functions of Gal-3 can be divided into both pro-inflammatory and anti-inflammatory effects [18,19]. Gal-3 within the cell is involved in innate immune-mediated inflammatory response by acting as inflammatory signaling molecule. Extracellularly secreted Gal-3 directly binds to LPS to decrease the production of pro-inflammatory cytokines after LPS stimulation, thereby exerting its anti-inflammatory effect [20]. This study suggested that the inhibition of Gal-3 attenuates neuroinflammation in SCI model. Lerman et al. [21] showed that Gal-3 exacerbates microglial activation and accelerates disease progression of chronic motor neuron degeneration.

NLRP3 is an important protein to recognize pathogens of the immune system in the body. It can be activated under the induction of ATP, virus and sodium urate to promote the binding of C-terminal LRR with its ligand, thereby promoting the complex formation of ASC and Caspase-1 to activate Caspase-1 [22]. In addition, it also promotes the mature and extracellular secretion of IL-1β to trigger inflammatory responses, thereby exerting a pathogenic effect in multiple systems and organs [23]. TXNIP is an important member of the body’s antioxidant stress response thioredoxin, mainly playing an oxidative stress role [24]. Recent studies have found that TXNIP can bind to and activate NLRP3 in a diseased state, thereby activating NLRP3 signaling pathway [22,24]. Our present data showed that Gal-3 regulates TXNIP/NLRP3 signaling pathway to promote neuroinflammation in SCI. The inhibition of TXNIP or NLRP3 reduced the effect of Gal-3 on neuroinflammation *in vitro* model. Simovic Markovic et al. [25] showed pro-inflammatory role of Gal-3 in acute colitis via NLRP3 in macrophages.

**Figure 9:**
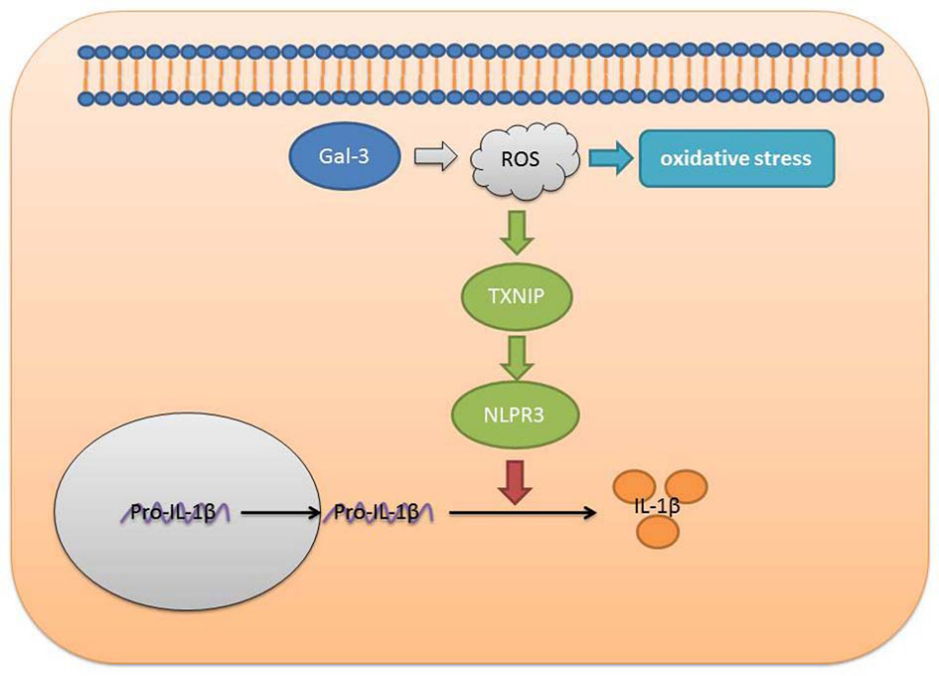
Gal-3 acts as a potential biomarker for SCI and Gal-3 deficiency, attenuates neuroinflammation through ROS/TXNIP/NLRP3 signaling pathway

In conclusion, the present study demonstrated that Gal-3 promoted neuroinflammation in SCI model by ROS/TXNIP/NLRP3 signaling pathway (Figure 9). Gal-3 may be a potential therapeutic strategy for SCI. Nevertheless, only PC12 cell line was employed in the present study. The preliminary findings obtained from the present study remain to be elucidated by a larger group of different cell lines.

## Ethic Approval

The present study was approved by the Scientific Review Committee and the Institutional Review Board of the First Affiliated Hospital of Xinjiang Medical University.

## Abbreviations

Gal-3: Galectin-3
LPS: lipopolysaccharide
ROS: reactive oxygen species
SCI: spinal cord injury
